# Community Groups Co-Design Evidence-Based Docudramas to Communicate About Child Spacing in Bauchi State, Nigeria: A Qualitative Descriptive Study

**DOI:** 10.1177/2752535X231221594

**Published:** 2023-12-12

**Authors:** Umaira Ansari, Khalid Omer, Yagana Gidado, Muhd Chadi Baba, Adamu Ibrahim Gamawa, Lois Ezekiel Daniel, Neil Andersson, Anne Cockcroft

**Affiliations:** 1 Centro de Investigación de Enfermedades Tropicales, 341132 Universidad Autónoma de Guerrero, Acapulco, Mexico; 2 Federation of Muslim Women’s Associations of Nigeria, Bauchi, Nigeria; 3 Bauchi State Primary Health Care Development Agency, Bauchi, Nigeria; 4 CIET-PRAM, Department of Family Medicine, 5620 McGill University, Montreal, Canada

**Keywords:** participatory research, community co-design, docudramas, short birth interval, family planning, maternal health, Nigeria

## Abstract

In Bauchi State, northern Nigeria, communities recognise short birth interval (*kunika* in the Hausa language) as harmful, but family planning is a sensitive topic. This paper describes the development of a culturally safe way to communicate about *kunika* in a conservative Muslim setting. The objective was to co-design culturally safe communication material, based on local knowledge about short birth interval, to share with women and men in households.

Six community co-design groups of women and six of men (total 96 participants) reviewed summaries of their previously created maps of perceived local causes of *kunika,* categorised as frequent sex, family dynamics and non-use of contraception. They advised how these causes could be discussed effectively and acceptably with women and their husbands in households and suggested storylines for three short video docudramas about the prevention of *kunika.* The research team created the docudramas with a local producer and fieldworkers piloted their use in households.

The design groups advised that communication materials should focus on child spacing rather than on limitation of family size. Even sensitive issues could be covered. People would not change their sexual behaviour but could be advised to use contraceptives to prevent *kunika*. The groups approved the final videos and six focus groups of visited women and men reported they were acceptable and helpful. Community co-design of communication about *kunika* was feasible and led to videos about a sensitive topic that were acceptable to ordinary men and women in communities in Bauchi.

## Background

From the 1950s to the 1990s, international funding bodies and governments strongly promoted family planning in low- and middle-income countries, yet fertility levels remained high.^1^ From the mid-1990s, the lexicon and focus shifted from reducing fertility towards reproductive health, promoting contraceptive use to protect maternal health, providing women with increased reproductive choices^2^ and supporting women’s rights to control their fertility.^3,4^ Short birth interval is associated with adverse health outcomes for children^5^ and probably for mothers.^6,7^ The World Health Organization recommends a birth interval of not less than 33 months, or 24 months between a birth and a subsequent pregnancy.^8^

Social and cultural factors may reduce the uptake of family planning in sub-Saharan Africa;^9^ some reports suggest Muslim communities reject the use of modern contraceptives.^10,11^ Policies and communication around family planning need to address cultural diversity.^12^ Informal discussions with women and men suggested acceptability and good recall of messages about healthy timing and spacing of pregnancy in a project working with religious leaders and a local NGO in Kano State, northern Nigeria.^13^ Nevertheless, how to communicate about family planning or birth spacing in an intercultural context remains poorly understood.

The Primary Health Care Development Agency (PHCDA), the government agency responsible for primary health care services in Bauchi State, Nigeria, collaborated with the NGO Federation of Muslim Women’s Associations of Nigeria (FOMWAN) Bauchi Chapter, and CIET-PRAM in McGill University in a successful trial of universal home visits to pregnant women and their spouses. The home visitors shared local survey evidence^14^ about risks for maternal and child health and encouraged action at household level.^15–18^ Concerned about the frequent pregnancies evident among women in the trial, the State PHCDA suggested creating a module about avoiding frequent pregnancies to include in the home visits. Initial discussions with health policy makers and religious leaders in Bauchi about addressing family planning as an issue led to a focus on short birth interval (*kunika* in the Hausa language). The concept of *kunika* means becoming pregnant before the last child is weaned. Islamic scholars encourage breast feeding for up to 24 months and discourage *kunika* because of the health risks to mothers and children.^19^ Focus groups of women and men in Bauchi understood the concept of *kunika* as being becoming pregnant while breastfeeding. Some groups further clarified that a further pregnancy should be delayed for a certain number of months or until the existing child could walk a certain distance. They were clear that *kunika* is a bad thing with adverse health, social, and financial consequences.^20^

There is evidence that community and mass media edutainment can improve health outcomes^21–23^ and studies have reported improvements in health knowledge and behaviours associated with the use of video edutainment as part of home visits interventions.^24,25^ In our participatory project^26^ about *kunika*, we aimed to create video edutainment docudramas to stimulate discussion of *kunika* within home visits. Docudramas are a hybrid of documentary and drama and are “fact-based representations of real events”^27^ (p. 480). In our work, a docudrama refers to a drama based on real local evidence. The project used integrated knowledge translation and exchange;^28^ knowledge users were involved throughout the process, from defining the concern to creating the video communication materials for sharing the findings. This paper describes the involvement of community co-design groups to review summaries of local knowledge about causes of *kunika* and to advise how to discuss these perceived causes in households, including in three short video docudramas. The docudramas, to be shown individually to women and men in household visits, were intended to spark discussion leading to actions in the households. The paper includes an early evaluation of the resulting three short video docudramas.

## Methods

### Setting

Bauchi State in Northeast Nigeria has a population of five million people, mostly Muslims of Hausa ethnicity. Families are large, and polygamy is common. In Bauchi, 63% of women have no formal education, 27% own a mobile phone, 19% participate in decisions about their own healthcare, and 46% can refuse sex with their husbands.^29^ Nigeria’s maternal mortality ratio of 1,047 is among the highest in the world^30^ and the situation is worse in Bauchi State.^31^ Only 33% of Bauchi women receive any antenatal care and 22% give birth attended by a formal skilled provider.^29^ Only 7% of currently married Bauchi women aged 15-49 years use any method of contraception.^29^ The median birth interval in Bauchi State is short at 30.9 months and the fertility rate is high at 7.2 children per woman.^29^ Nigerian soap-operas are very popular in Bauchi State, and we have previously used soap-opera style docudramas as edutainment in Bauchi.^15^

### Overview of Methods and Research Partnership

Fieldworkers discussed with community co-design groups summaries of maps these groups had previously created to depict their knowledge of perceived causes of *kunika* (short birth interval) and asked them about communicating about these causes of *kunika* with households. Based on the proposals of the co-design groups, the research team created three video docudramas about *kunika*. To evaluate the videos, we sought the views of half the co-design groups about them. Home visitors then used the videos in home visits in three wards and we conducted six focus groups with women and men from visited households to hear their views about the videos.

The research partnership was the same as for the earlier trial of home visits to promote maternal and child health described in the Background and has been working together for 15 years. Local team members themselves facilitated and reported the discussions. Traditional leaders in each community actively supported the work and nominated group participants.

### Evidence Presented to Community Co-Design Groups

The evidence came from fuzzy cognitive mapping (FCM) undertaken in 2018.^32–34^ In the FCM exercise, separate groups of women and men in 12 randomly selected urban, rural, and rural-remote communities in Toro local government area (LGA) created 48 maps of their perceptions about the causes and protective factors for *kunika.* An article describes the FCM process in detail.^35^ Briefly, each group mapped their knowledge of the causes of *kunika*. They used arrows to show how the causal factors linked to each other and to *kunika* and weighted the strengths of the links between 5 (strongest) and 1 (weakest).

The research team produced summary maps derived from analysis of the cognitive maps of the most influential perceived causes of *kunika.* They included:

1. Frequent sex and related factors (such as use of aphrodisiacs and pornography, husband and wife sharing a bed, women dressing to entice men, high desire, resuming sex while still breastfeeding, and being impatient or careless)
2. Family dynamics (such as pressure to have more children and competition between co-wives)
3. Non-use of contraception including modern methods (such as injections, pills, intrauterine devices, and condoms) and traditional methods (withdrawal, calendar methods)

**Figure 1.**
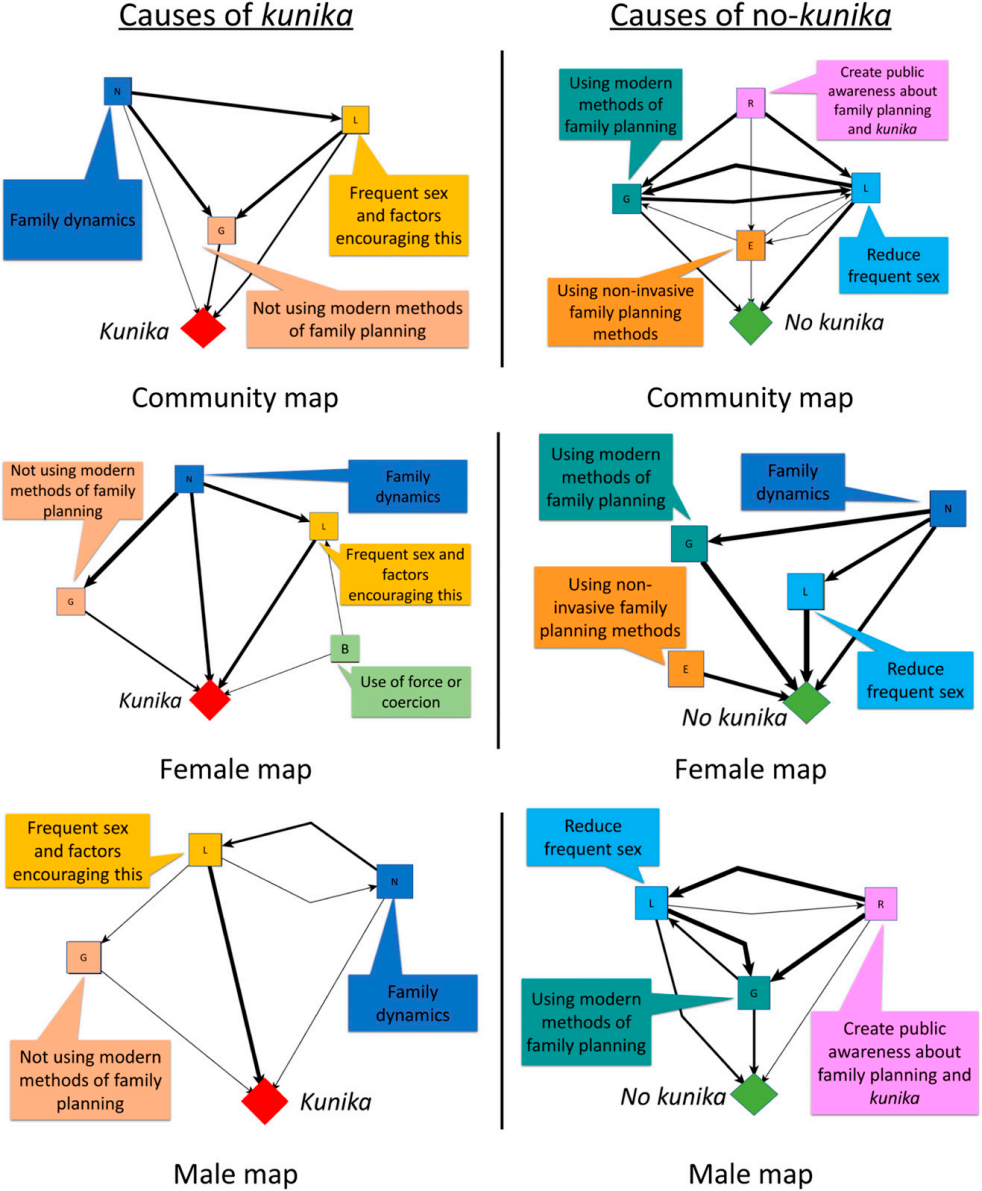
Summary maps for causes and prevention of *kunika*.

These summary maps were the starting point for discussions with community co-design groups about how to talk to households about causes and prevention of *kunika*. Figure 1 shows the summary maps for causes and prevention of *kunika* for all community groups and from men’s and women’s groups separately.

### Co-design Group Discussion Guide

The research team developed a draft discussion guide and piloted it with a women’s and a men’s group in one community (see supplementary file 1). The first section of the guide presented the summary cognitive maps, reminding participants about the causes of *kunika* they had identified in mapping sessions, checking their agreement with the categorization of these causes, and discussing differences between men’s and women’s maps. The second section sought suggestions about how to talk to households about these causes. A final section invited suggestions for storylines and dialogue for short video docudramas about the causes and prevention of *kunika*.

### Implementation of the Co-Design Groups

The group discussions took place in 2019 in the six wards of Toro LGA where we implemented home visits to pregnant women and their spouses.^15^ We randomly selected six communities (two urban, two rural, and two rural-remote) among the 12 randomly selected communities where groups had previously created cognitive maps. Separate co-design groups of women and men took place in each community, a total of 12 groups, each with seven to nine participants. The community leaders nominated the group participants, all of whom had previously created fuzzy cognitive maps in their communities. They also identified suitable neutral and private venues for the group meetings, often the school.

All the group participants were married Muslims. The average age of the 47 women was 39 years, while the average age for the 49 men was 49 years. Nearly all the men (80%) had some formal education, as did more than half (59%) of the participating women. The level of formal education among the women was higher than the 37% across the whole of Bauchi State,^29^ but similar to the 53% (12,308 out of 23,944) among pregnant women registered in the home visits programme in Toro LGA.

The local research team facilitated group discussions; women facilitated groups of women and men facilitated groups of men. All the facilitators were college graduates with experience of conducting community group discussions. For each group, a local researcher acted as reporter and took detailed notes of the discussion, including writing quotes as nearly verbatim as possible, and describing group dynamics. We did not audio-record the sessions. The discussions lasted about 3 hours, including a refreshment break, and were in the Hausa language. We provided refreshments and a small transport allowance to participants. Women participants received their allowance directly. All group discussions took place in private settings, with women’s and men’s groups in separate locations in the site. Participants undertook not to share what others had said during the session. Reports from the groups did not identify any individuals or communities.

The facilitator and reporter together finalised the report of each group on the same day, including translating the report into English.

### Creation of the Video Docudramas

UA reviewed suggestions from the co-design groups about how to talk about the different causes of *kunika,* and their proposed storylines and dialogues. She drafted scripts for three docudramas around the themes of family dynamics, frequent sex, and non-use of contraception, incorporating storylines and dialogues proposed by the co-design groups. Members of the local research team helped to finalize the scripts.

A local Bauchi producer hired local actors and filmed the dramas, each about 4 minutes long, in a community typical of Toro LGA. The actors wore typical local dress and spoke in the Hausa language. The local research team monitored the filming and UA worked with the producer on post-production. Government officers from the Ministry of Health and PHCDA and the local research team reviewed the first cut of the videos. UA and the producer incorporated their suggestions into the final cut.

### Evaluation of the Video Docudramas

We evaluated the videos in two ways. First, the research team returned to six of the 12 co-design groups in three communities (three women’s and three men’s groups), showed them the videos, and sought their views about them. To support a negative case analysis,^36^ the facilitators probed for negative views about each video. Second, in two wards of Toro LGA, home visitors^15^ included a module about *kunika* in their home visits to women and their spouses after the birth of a child, showing the three *kunika* video docudramas on their cell phones. They showed the *kunika* videos in 2936 households. About 1 year later, the research team convened three focus groups of visited women (total 30 women) and three focus groups of visited male spouses (total 30 men) who had been shown the *kunika* videos to talk about their recall of the videos, whether they had discussed them with others, and what they thought about them. The facilitators specifically asked participants about anything they did not like about the videos, anyone they knew of who had not liked the videos and why, and what else would be useful to include. We did not incorporate community members’ comments and feedback in the videos.

## Results

This section first describes the views of the co-design groups about how to talk to women and men in households about causes and prevention of *kunika* and their suggestions for the videos. It then describes the results of the evaluation of the videos by (a) the co-design groups and (b) the women and men shown the videos in household visits.

### Views From the Co-Design Groups

Participants in the co-design groups recalled making cognitive maps and the factors they perceived as causes of *kunika*. They found the summary maps easy to relate to. Considering differences between women’s and men’s maps, women’s groups suggested women more often mentioned forced or coerced sex as a cause of *kunika* because men considered they had a right to have sex with their wives or did not consider they were wrong to use force. Men’s groups suggested men did not see anything wrong with forcing their wives or kept silent because they are the ones at fault.

Groups noted that communication materials should avoid the Hausa term *tsarin iyalin* (family planning) because many people conflate it with the term *kayyade iyali* (limiting the size of the family), which is unacceptable, unlike *tazarar haihuwa* (spacing births), which is desirable.

#### How to Talk About Frequent Sex

The groups advised it was possible to discuss sensitive topics related to frequent sex, such as the use of aphrodisiacs and pornography (reportedly common in Bauchi), women dressing provocatively to entice their spouses, high sexual desire of both men and women, couples being impatient and careless, and even forced or coerced sex. But they laughed at the idea that people would accept advice to have sex less frequently. They proposed reminding people that frequent sex would lead to *kunika,* but they could avoid this by using effective contraception. Although women identified forced sex as a cause of *kunika* in cognitive maps, this topic did not feature in the suggestions of the co-design groups about messages to prevent *kunika*, even in women’s groups.

“You can say: ‘a man can love and get attracted to his wife, and putting on tight clothes and make-up is good, but you should know it leads to *kunika* and the breastfeeding baby suffers’.” (Men’s group participant)

“Truth be told, you cannot stop people from using sex drive medicine. People should use it, but they should also use protection to avoid *kunika*.” (Women’s group participant)

#### How to Talk About Family Dynamics

Competition between co-wives, and men’s role in causing this, was a prominent factor within family dynamics. Participants suggested advising both men and women they could still have a large family, with adequate spacing between births, assisted by using contraception. The groups suggested that husbands and wives should ignore social and family pressure to have many children.

“Most men do not treat their wives equally. This means that some wives will do *kunik*a to get gifts.” (Men’s group participant)

“People wanting a large family should use contraception to space their children to have a healthy and large family.” (Women’s group participant)

“Tell them [men]: ‘Just ignore people’s pressure and consider your own affairs’.” (Men’s group participant)

#### How to Talk About Using Contraceptives

Group participants did not think it would be difficult to talk about using contraceptives. They suggested stressing the benefits of using contraceptives to achieve adequate child spacing. Many groups recommended advising women and men to seek advice from health workers to find the best method of contraception for them and avoid side-effects.

“I would start by explaining the effect of *kunika* on the health of the woman and the child. I would then talk about the economic condition of the family, before advising them to use contraceptives.” (Men’s group participant, explaining how to talk to men)

“I would tell them to get advice from a health worker because modern methods of contraception can have side-effects and that is what makes people avoid using contraception.” (Women’s group participant)

#### Storylines and Dialogues for the Video Docudramas

Asked to suggest storylines, some of the co-design groups, especially women’s groups, proposed specific storylines, and spontaneously role-played potential dialogues between characters.

“There are two wives in one house - one with many young children and the other with few children. The husband comes home with a shopping bag. The woman with many young children takes the bag to show everything belongs to her because she has many children. The other woman notes the problems of having many children of similar age and feels happy to be independent with a good business and able to take care of her children. These topics emerge in discussion between the second woman and her friend.”

Other groups gave more generic suggestions, such as showing dirty households with many young children because of *kunika*. The three resulting docudramas (on family dynamics, frequent sex and use of contraceptives) all featured women and men struggling in some way because of *kunika*, and other characters advising them about how they could prevent *kunika*, through addressing aspects of family dynamics and using contraception to avoid *kunika* resulting from frequent sex.

The three video docudramas, covering family dynamics, frequent sex, and use of contraceptives, with English subtitles, are available online.^37^

### Evaluation of the Videos

#### Co-Design Groups

Co-design group participants voiced strong approval of the videos. They said the dramas reflected their views and they felt proud to have played a useful role in their communities.

“We made these videos! It is a great feeling to contribute to positive change in our community.” (Women’s group)

Probed for negative comments and suggestions, some groups suggested depicting involvement of men more strongly. Some groups suggested more depiction of the adverse consequences of *kunika* and the benefits of avoiding *kunika*.

“Show more contraceptive methods for men [such as condoms]. The men should be the ones using the contraceptives, not women.” (Women’s group)

“Show that Adamu and Aisha have stopped doing *kunika* and they have a better, healthy and happy family in the end” (Women’s group)

#### Focus Groups of People Who Had Seen the Videos in Household Visits

Focus group participants recalled most of the contents of the videos and the discussion about them with the home visitors. They were generally very positive about the videos. They reported discussing the videos with their families, who mostly reacted positively. Some said the videos stimulated them to act.

“The videos and discussions have made many people take positive action and plan their family by using contraceptives to avoid *kunika*.” (Woman participant)

“My mother in-law liked the idea very much. According to her, times are changing, *kunika* is becoming a thing of the past and it is good to move with the times.” (Woman participant)

“Seeing the videos made me visit the health facility for child spacing advice.” (Woman participant)

When pressed for negative views, some people said their friends or family did not support the videos because they did not agree with the idea of preventing *kunika*.

“My mother in-law was not in support of the videos because she thinks they will stop me from having children.” (Woman participant)

## Discussion

Community co-design groups of men and women in Bauchi State suggested culturally appropriate ways to talk about the main local causes of *kunika* they had previously identified. Their recommendations led to development of three video docudramas which were well-received by women and men in households. The strong sense of ownership of the videos and pride in their contribution to their communities described by members of the co-design groups reflect the transformative impact that participatory research can have on participants.^26,38^

The videos were based on local evidence. Our participatory approach of Socializing Evidence for Participatory Action (SEPA) proposes that communities are motivated to make informed decisions and act when they hear about local evidence.^39,40^ We have used SEPA in diverse settings;^41–44^ the home visits in Bauchi shared local evidence about risk factors for maternal and child health.^14^ Some authors recommend cultural adaptation of health communication materials developed elsewhere.^45,46^ But the *content* of communication materials might be quite different depending on local evidence. The videos we created based on local knowledge in Bauchi could be useable in other states in northern Nigeria but probably not in the different cultural settings of southern Nigeria or other countries. However, the *process* of using local evidence to develop communication materials is widely applicable.

Studies report lower contraceptive prevalence among women in Muslim communities.^11,47,48^ Clumsy and culturally insensitive attempts to promote use of modern contraception in conservative Muslim cultures have led to negative connotations of “family planning” as an externally driven, unwelcome concept.^49,50^ Islam encourages birth spacing to protect the health of mothers but prohibits family planning to limit the number of births.^19^ *Kunika* is a well-known concept in Bauchi and focus groups confirmed *kunika* is considered a bad thing.^20^ Our videos focused on *kunika* and its prevention and, as advised by the co-design groups, introduced the concept of contraception in the context of preventing *kunika*.

Originally proposed in New Zealand,^51^ cultural safety requires “a space that is spiritually, socially, emotionally and physically safe for people; where there is no assault, challenge or denial of their identity, of who they are, and what they need.”^52^ We believe our research around the problem of *kunika* in Bauchi led to communication materials congruent with the religious and cultural context, and supported cultural safety when addressing *kunika* in home visits.

Participatory research aspires to engage knowledge users throughout the research process, including communication of results.^26^ Nevertheless, the task of creating communication materials often relies on specialists. But intended users can be engaged to co-design or adapt health communication materials^53,54^ including co-design of videos.^55^ In Bauchi, the co-design group suggestions led to the video scripts, and a local Bauchi production team filmed the videos, with support and advice from members of the local research team. The depiction of people like themselves, in living circumstances they recognised, may have made it easier for people viewing the videos to accept their messages. Studies of the use of videos in community interventions report that videos work well when people can identify with characters and story lines.^56,57^

Community participation in research can come with difficult tensions, including ethical issues.^58^ Community members may describe local traditions and norms or propose local solutions that feel uncomfortable to researchers from different cultures. Although women had identified forced or coerced sex as an important cause of *kunika* in their cognitive maps, the same women in co-design groups did not propose talking about forced or coerced sex when communicating about *kunika*. The research team in our study did not press participants on this issue. It could be important to explore this specifically in a future study.

### Limitations

The evidence used for co-design of the docudramas was the knowledge of female and male community members about causes of *kunika*, systematized through FCM. The causes of an outcome surfaced in fuzzy cognitive maps are not necessarily “true” causes, in the sense that they predict outcomes, but they are causes from the perspective of the map authors and relevant to co-design of communication materials in a participatory approach. The local knowledge of perceived causes of *kunika* was congruent with findings from previous qualitative studies in low- and middle-income countries and, although the overlap of studied associations was limited, it did not conflict with findings of published quantitative studies.^59^ Persson et al^60^ consider that local knowledge (or practical experience) is valid evidence, despite its limitations, and its integration with scientific evidence can avoid problems of evidence failing to answer the right questions for local application. The design group participants were nominated by community leaders and might have been more articulate than average community members, as well as rather older. Their views might differ from those of younger community members.

We did not audio-record the discussions of the groups. We do not believe this led to missing important information. We agree with Rutakumwa et al that the taking of detailed notes by well-trained field workers is an effective way of reporting focus group discussions and in-depth interviews and sometimes better than audio-recording.^61^

Intended for use in household visits, the videos focussed on causes of *kunika* actionable at household and community level. They did not cover supply-side issues, such as lack of contraceptives in facilities, although these were identified in the cognitive maps as a cause of non-use of contraception and need to be addressed by the government health services. The extent of the co-design of the videos was limited in that the co-design groups did not co-create the videos and their feedback about the videos came too late to modify content. We continue to test approaches to increase engagement of local stakeholders in creation of communication materials in Bauchi. A current project includes support for youth to create their own cellphilms (1-5 min videos filmed on cell phones)^62^ depicting their sexual and reproductive health concerns and solutions.^63,64^

## Conclusion

Community co-design of videos about prevention of *kunika*, based on local knowledge about its causes, was feasible and led to videos about a sensitive topic that were acceptable to ordinary men and women in communities in Bauchi. The incorporation of local knowledge and views into the videos made them more accessible for local audiences. Future research needs to evaluate the impact of the videos and discussion guide on contraceptive intention and use, and on birth spacing. Participatory research has a useful role in culturally safe co-design of evidence-based communication materials.

## Supplemental Material

Supplemental Material - Community Groups Co-Design Evidence-Based Docudramas to Communicate About Child Spacing in Bauchi State, Nigeria: A Qualitative Descriptive Study Supplemental Material for Community Groups Co-Design Evidence-Based Docudramas to Communicate About Child Spacing in Bauchi State, Nigeria: A Qualitative Descriptive Study by Umaira Ansari, Khalid Omer, Yagana Gidado, Muhd Chadi Baba, Adamu Ibrahim Gamawa, Lois Ezekiel Daniel, Neil Andersson, and Anne Cockcroft in Community Health Equity Research & Policy

## References

[1] Bongaarts J Mauldin WP Phillips JF . The demographic impact of family planning programs. Stud Fam Plann 1990; 21(6): 299–310. 2075620

[2] United Nations Population Fund . Programme of action of the international Conference on population development 20th Anniversary Edition. New York: United Nations Population Fund, 2014. ISBN 978-0-89714-022-5, unpd_workshop_201907_icpd_programme_of_action_en.pdf (accessed 10 December 2023).

[3] World Medical Association . Statement on family planning and the right of a woman to contraception. 2020, https://www.wma.net/policies-post/wma-statement-on-family-planning-and-the-right-of-a-woman-to-contraception/ (accessed 10 December 2023).

[4] Prata N Fraser A Huchko M , et al. Women’s empowerment and family planning: a review of the literature. J Biosoc Sci 2017; 49(6): 713–743. DOI: 10.1017/S0021932016000663. 28069078 PMC5503800

[5] Conde-Agudelo A Rosas-Bermudez A Kafury-Goeta AC . Birth spacing and risk of adverse perinatal outcomes: a meta-analysis. JAMA 2006; 295: 1809–1823. DOI: 10.1001/jama.295.15.1809. 16622143

[6] Rutstein SO . Effects of preceding birth intervals on neonatal, infant and under-five years mortality and nutritional status in developing countries: evidence from the demographic and health surveys. Int J Gynaecol Obstet. 2005; 89: S7–S24. doi:10.1016/j.ijgo.2004.11.012. 15820369

[7] Dewey KG Cohen RJ . Does birth spacing affect maternal or child nutritional status? A systematic literature review. Matern Child Nutr 2007; 3: 151–173. DOI: 10.1111/j.1740-8709.2007.00092.x. 17539885 PMC6860904

[8] World Health Organization . Report of a WHO technical consultation on birth spacing. Geneva, Switzerland: World Health Organization, June 2005, https://apps.who.int/iris/handle/10665/69855 (accessed 3 September 2023).

[9] Haider TL Sharma M . Barriers to family planning and contraception uptake in Sub-Saharan Africa: a systematic review. Int Q Community Health Educ 2013; 33(4): 403–413, DOI: 10.2190/IQ.33.4.g. 24044930

[10] Sule ST Umar HS Madugu NH . Knowledge and use of modern contraception among Muslim women in Zaria, Nigeria. J Islam Med Assoc 2006; 38: 10. DOI: 10.5915/38-1-5293.

[11] Mishra V . Muslim/non-Muslim differentials in fertility and family planning in India. Population and Health Series 2004; 112: 1, https://scholarspace.manoa.hawaii.edu/handle/10125/3749 (Accessed 3 September 2023).

[12] Obono O . Cultural diversity and population policy in Nigeria. Popul Dev Rev 2003; 29(1): 103–111, DOI: 10.1111/j.1728-4457.2003.00103.x.

[13] Lane C Joof YM Hassan AA , et al. Promoting healthy timing and spacing of pregnancy with young married women in Northern Nigeria: a short report. Afr J Reprod Health 2012; 16(2): 263–269, https://www.ajol.info/index.php/ajrh/article/view/77853 22916558

[14] Andersson N Omer K Caldwell D , et al. Male responsibility and maternal morbidity: a cross-sectional study in two Nigerian states. BMC Health Serv Res 2011; 11(supp2): S7. DOI: 10.1186/1472-6963-11-S2-S7. 22375828 PMC3332566

[15] Cockcroft A Omer K Gidado Y , et al. Impact of universal home visits on maternal and infant outcomes in Bauchi state, Nigeria: protocol of a cluster randomized controlled trial. BMC Health Serv Res 2018; 18: 510, DOI: 10.1186/s12913-018-3319-z. 29970071 PMC6029180

[16] Cockcroft A Omer K Gidado Y , et al. The impact of universal home visits with pregnant women and their spouses on maternal outcomes: a cluster randomised controlled trial in Bauchi State, Nigeria. BMJ Glob Health 2019; 4: e001172. DOI: 10.1136/bmjgh-2018-001172. PMC6407530 30899560

[17] Omer K Joga A Dutse U , et al. Impact of universal home visits on child health in Bauchi State, Nigeria: a stepped wedge cluster randomised controlled trial. BMC Health Serv Res 2021; 21: 1085, DOI: 10.1186/s12913-021-07000-3. 34641865 PMC8513291

[18] Cockcroft A Omer K Gidado Y , et al. Universal home visits improve male knowledge and attitudes about maternal and child health in Bauchi State, Nigeria: secondary outcome analysis of a stepped wedge cluster randomised controlled trial. J Glob Health 2022; 12: 04003. DOI: 10.7189/jogh.12.04003. 35136595 PMC8818298

[19] Renowned Islamic Scholars , Ulema, and Medical Professionals. Islamic perspectives on reproductive health and childbirth spacing in Nigeria. Abuja: Nigerian Urban Reproductive Health Initiative, 2017, https://tciurbanhealth.org/wp-content/uploads/2018/12/islamic_perspective_new.pdf (accessed 10 December 2023).

[20] Ansari U Pimentel J Omer K , et al. *Kunika* women are always sick”: views from community focus groups on short birth interval (*kunika*) in Bauchi State, Northern Nigeria. BMC Wom Health 2020; 20(113): 113. 10.1186/s12905-020-00970-2 PMC7245922 32448373

[21] Shen F Han J . Effectiveness of entertainment education in communicating health information: a systematic review. Asian J Commun 2014; 24: 605–616. DOI: 10.1080/01292986.2014.927895.

[22] Orozco-Olvera V Shen F Cluver L . The effectiveness of using entertainment education narratives to promote safer sexual behaviors of youth: a meta-analysis, 1985-2017. PLoS One 2019; 14(2): e0209969, DOI: 10.1371/journal.pone.0209969. 30753185 PMC6372167

[23] Sood S Henderson Riley A Alarcon K . Entertainment-education and health and risk messaging. Oxford Research Encyclopedia of Communication. Oxford University Press. https://oxfordre.com/communication/view/10.1093/acrefore/9780190228613.001.0001/acrefore-9780190228613-e−245 (accessed 10 December 2023).

[24] Ayala GX Ibarra L Horton L , et al. Evidence supporting a promotora-delivered entertainment education intervention for improving mothers’ dietary intake: the Entre Familia: Reflejos de Salud Study. J Health Commun. 2015; 20: 165–176. doi:10.1080/10810730.2014.917747. 25375276

[25] Dway NS Soonthornworasiri N Jandee K , et al. Effects of edutainment on knowledge and perceptions of Lisu mothers about the immunisation of their children. Health Educ J 2016; 75(2): 131–143. DOI: 10.1177/0017896915569086.

[26] Andersson N . Participatory research—a modernizing science for primary health care. J Gen Fam Med 2018; 19: 154–159, DOI: 10.1002/jgf2.187. 30186727 PMC6119794

[27] Ogunleye F . Television docudrama as alternative records of history. Hist Afr 2005; 32: 479–484, DOI: 10.1353/hia.2005.0019.

[28] Kothari A Wathen CN . Integrated knowledge translation: digging deeper, moving forward. J Epidemiol Community Health 2017; 71: 619–623, DOI: 10.1136/jech-2016-208490. 28298415

[29] National Population Commission - NPC and ICF . Nigeria Demographic and health survey 2018 - final report. Abuja, Nigeria: NPC and ICF. (accessed 3September 2023) https://dhsprogram.com/pubs/pdf/FR359/FR359.pdf

[30] Trends in maternal mortality 2000 to 2020: estimates by WHO, UNICEF, UNFPA, world bank group and UNDESA/population Division. Geneva: World Health Organization, 2023. Available: https://www.who.int/publications/i/item/9789240068759 (Accessed 3 September 2023)

[31] Kabo I Otolorin E Williams E , et al. Monitoring maternal and newborn health outcomes in Bauchi State, Nigeria: an evaluation of a standards-based quality improvement intervention. Int J Qual Health Care 2016; 28(5): 566–572. 27512125 10.1093/intqhc/mzw083 PMC5105602

[32] Kosko B . Fuzzy cognitive maps. Int J Man Mach Stud 1986; 24: 65–75, DOI: 10.1016/S0020-7373(86)80040-2.

[33] Giles BG Findlay CS Haas G , et al. Integrating conventional science and aboriginal perspectives on diabetes using fuzzy cognitive maps. Soc Sci Med 2007; 64: 562–576. DOI: 10.1016/j.socscimed.2006.09.007. 17084952

[34] Andersson N Silver H . Fuzzy cognitive mapping: an old tool with new uses in nursing research. J Adv Nurs 2019; 75: 3823–3830, DOI: 10.1111/jan.14192. 31486102

[35] Sarmiento I Ansari U Omer K , et al. Causes of short birth interval (kunika) in Bauchi State, Nigeria: systematizing local knowledge with fuzzy cognitive mapping. Reprod Health 2021; 18: 74, DOI: 10.1186/s12978-021-01066-2. 33823874 PMC8022364

[36] Morse JM . Critical analysis of strategies for determining rigor in qualitative inquiry. Qual Health Res 2015; 25: 1212–1222. DOI: 10.1177/1049732315588501. 26184336

[37] https://www.youtube.com/playlist?list=PL17QiK-Lpq1xhmZrEZaIrVsiGu9Z6fO51

[38] Ozer EJ Douglas L . The impact of participatory research on urban teens: an experimental evaluation. Am J Community Psychol 2013; 51(2): 66–75. 22875686 10.1007/s10464-012-9546-2

[39] Andersson N . Building the community voice into planning: 25 years of methods development in social audit. BMC Health Serv Res 2011; 11(Suppl 2): S1, DOI: 10.1186/s12889-017-4287-7. 22376121 PMC3397387

[40] Ledogar RJ Arosteguí J Hernández-Alvarez C , et al. Mobilising communities for Aedes aegypti control: the SEPA approach. BMC Publ Health 2017; 17(Suppl 1): 403, DOI: 10.1186/s12889-017-4298-4. PMC5506583 28699561

[41] Omer K Mhatre S Ansari N , et al. Evidence-based training of frontline health workers for door-to-door health promotion: a pilot randomized controlled cluster trial with lady health workers in Sindh Province, Pakistan. Patient Educ Couns 2008; 72: 178–185. DOI: 10.1016/j.pec.2008.02.018. 18395396

[42] Andersson N Cockcroft A Ansari NM , et al. Evidence-based discussion increases childhood vaccination uptake: a randomised cluster controlled trial of knowledge translation in Pakistan. BMC Int Health Hum Rights 2009; 9(Suppl 1): S8, DOI: 10.1186/1472-698X-9-S1-S8. 19828066 PMC3226240

[43] Villegas-Arrizón A Andersson N Ledogar RJ . Micro-regional planning: evidence-based community buy-in for health development in five of Mexico’s poorest rural districts. BMC Health Serv Res. 2011; 11(Suppl 2): S2. doi:10.1186/s12889-017-4288-6. 22375532 PMC3332561

[44] Andersson N Nava-Aguilera E Arosteguí J , et al. Evidence based community mobilization for dengue prevention in Nicaragua and Mexico (Camino Verde, the Green Way): cluster randomized controlled trial. BMJ 2015; 351: h3267, DOI: 10.1136/bmj.h3267. 26156323 PMC4495677

[45] European Centre for Disease Prevention and Control . Translation is not enough – cultural adaptation of health communication materials. Stockholm: ECDC, 2016, https://www.ecdc.europa.eu/sites/portal/files/media/en/publications/Publications/translation-is-not-enough.pdf (accessed 3 September 2023).

[46] Isler J Sawadogo NH Harling G , et al. Iterative adaptation of a mobile nutrition video-based intervention across countries using human-centered design: qualitative study. JMIR Mhealth Uhealth 2019; 7(11): e13604, DOI: 10.2196/13604. 31710302 PMC6878105

[47] Agadjanian V Yabiku ST Fawcett L . History, community milieu, and Christian‐Muslim differentials in contraceptive use in sub‐Saharan Africa. J Sci Study Relig 2009; 48: 462–479, DOI: 10.1111/j.1468-5906.2009.01460.x.

[48] Abate M Tareke A . Individual and community level associates of contraceptive use in Ethiopia: a multilevel mixed effects analysis. Arch Publ Health 2019; 77: 46, DOI: 10.1186/s13690-019-0371-z. PMC6820945 31687139

[49] El Hamri N . Approaches to family planning in Muslim communities. J Fam Plann Reprod Health Care 2010; 36: 27–31. DOI: 10.1783/147118910790291019. 20067669

[50] Gwarzo TH . Islamic religious leaders and family planning in Northern Nigeria: a case study of Zamfara, Sokoto and Niger States. J Muslim Minority Aff 2011; 31: 143–151, DOI: 10.1080/13602004.2011.556894.

[51] Hill P . Cultural safety hui of Whanau Kawa Whakaruruhau, Apumoana Marae, Rotorua. Palmerston North N.Z: PSI Solutions, 1991, https://books.google.co.bw/books/about/Cultural_Safety_Hui_of_Whanau_Kawa_Whaka.html?id=ZQu4MQAACAAJ&redir_esc=y (Accessed 10 December 2023).

[52] Williams R . Cultural safety - what does it mean for our work practice? Aust N Z J Public Health 1999; 23(2): 213–224. DOI: 10.1111/j.1467-842x.1999.tb01240.x. 10330743

[53] Minian N Noormohamed A Zawertailo L , et al. A method for co-creation of an evidence-based patient workbook to address alcohol use when quitting smoking in primary care: a case study. Res Involv Engagem 2018; 4: 4, DOI: 10.1186/s40900-018-0086-2. 29445521 PMC5798180

[54] Hswen Y Bickham DS . Engaging African American teens in co-creating and disseminating social media based HIV prevention messages. J Hosp Manag Health Policy 2018; 2: 41, DOI: 10.21037/jhmhp.2018.07.06.

[55] Chávez V Israel B Allen AJ , et al. A bridge between communities: video-making using principles of community-based participatory research. Health Promot Pract 2004; 5(4): 395–403, DOI: 10.1177/1524839903258067. 15358912

[56] Desta BF Mohammed H Barry D , et al. Use of mobile video show for community behavior change on maternal and newborn health in rural Ethiopia. J Midwifery Wom Health 2014; 59(Suppl 2): S65–S72, DOI: 10.1111/jmwh.12111. 24588918

[57] Mutanda JN Waiswa P Namutamba S . Community-made mobile videos as a mechanism for maternal newborn and child health education in rural Uganda; a qualitative evaluation. Afr Health Sci 2016; 16(4): 923–928, DOI: 10.4314/ahs.v16i4.6. 28479882 PMC5398436

[58] Ledogar RJ Hernández-Alvarez C Morrison AC , et al. When communities are really in control: ethical issues surrounding community mobilisation for dengue prevention in Mexico and Nicaragua. BMC Publ Health 2017; 17(Suppl 1): 410. DOI: 10.1186/s12889-017-4305-9. PMC5506591 28699548

[59] Pimentel J Ansari U Omer K , et al. Factors associated with short birth interval in low- and middle-income countries: a systematic review. BMC Pregnancy Childbirth 2020; 20: 156, DOI: 10.1186/s12884-020-2852-z. 32164598 PMC7069040

[60] Persson J Johansson EL Olsson L . Harnessing local knowledge for scientific knowledge production: challenges and pitfalls within evidence-based sustainability studies. Ecol Soc 2018; 23(4): 38, DOI: 10.5751/ES-10608-230438.

[61] Rutakumwa R Mugisha JO Bernays S , et al. Conducting in-depth interviews with and without voice recorders: a comparative analysis. Qual Res 2020; 20(5): 565–581. 32903872 10.1177/1468794119884806 PMC7444018

[62] Thompson J Mitchell C Starr L . Cellphilming: a tool for addressing gender equality—facilitators’ guide. Ottawa, ON: CODE, 2019, https://www.mcgill.ca/morethanwords/files/morethanwords/cellphilm_guidebook.final_.pdf (Accessed 3 September 2023).

[63] Cockcroft A Omer K Gidado Y , et al. Impact-oriented dialogue for culturally safe adolescent sexual and reproductive health in Bauchi state, Nigeria: protocol for a co designed pragmatic cluster randomized controlled trial. JMIR Res Protoc 2022; 11(3): e36060. DOI: 10.2196/36060. 35289762 PMC8965671

[64] Cockcroft A Ansari U Omer K , et al. Getting ready to cellphilm: training the team to work with adolescents to create cellphilms to support intergenerational dialogue in Bauchi State, Nigeria. In: Mitchell C Sadati SMH Starr LJ Roy S (ed) Revisioning cellphilming. New York: Springer Nature, 2023. (in press).

