# Supplementary material for: Community Groups Co-Design Evidence-Based Docudramas to Communicate About Child Spacing in Bauchi State, Nigeria: A Qualitative Descriptive Study
Source: Community Health Equity Res Policy. 2023 Dec 12;45(1):35–44. doi: 10.1177/2752535X231221594 (PMC11418317; doi:10.1177/2752535X231221594)
Supplement: Supplemental Material - Community Groups Co-Design Evidence-Based Docudramas to Communicate About Child Spacing in Bauchi State, Nigeria: A Qualitative Descriptive Study [file sj-pdf-1-qch-10.1177_2752535X231221594.pdf]

Supplementary File 1: Field Guide for Dialogue Groups

Hello and Greetings,

My name is [*mention your name*], and this is [*mention name of reporter*]. **We work with FOMWAN and CIET, who are working together with the Bauchi State government and Toro LGA**. As many of you already know, male and female home visitors from our group have been **visiting households** here and talking to pregnant women and their husbands about ways to protect the health of pregnant women and their babies.

Many people are concerned that ***kunika* can be bad for the health of a woman and her children**, as well as putting an increased financial burden on the household. As you know, we call it *kunika* when a woman becomes pregnant again while she is still breastfeeding her previous child.

Last year we sat with you to create **maps of your knowledge about causes of *kunika* and what can help to prevent it.** We made those maps on a white board. We put your knowledge about what **causes or prevents *kunika* on the map (on tiles)** and then you drew **arrows to show how these things linked together** to cause or prevent *kunika***,** and marked how **strong each link was**. People in other communities made maps as well: overall **26 groups made maps** about causes and prevention of *kunika*.

We have **combined the information from all these maps** and today we would like to **share** these findings with you. We would also like to ask for your **views about how to include issues about kunika in the household visits to pregnant women and their husbands**. We expect that our meeting will take **less than two hours**. At the end we will provide some light **refreshments**.

Our discussion is **confidential**. We are going to make notes, but we will not include any names. We will share findings with other people – like health planners – but without any names. If we publish the findings they will not include any information to identify you or your community.

Of course, many of you already know one another. Please **respect the confidentiality** of our discussion and don't mention outside of this group any views shared in the group. We will not ask you about any personal experiences, but some people might decide to share personal experiences: please don't repeat their stories outside this group.

Your participation today is entirely **voluntary** and you can leave the session at any time if you feel uncomfortable or if you have other things you need to do. Thank you very much, in advance, for your time today.

Part 1
Sharing the evidence

1. Do you remember some of the factors you all identified when we made the map on Kunika?

<Facilitator mentions the following>
For the causes of Kunika, all the groups identified
Frequent sex
Family dynamics

Most groups mentioned
Not using modern methods of family planning
Lack of awareness on FP and fear of their side-effects

Many groups also mentioned
Not using non-invasive family planning methods

Many groups also identified
Not using health services
Belief in faith or fate
Use of force or coercion
Lack of male involvement
Socio-economic conditions and
Fertility including fertility after giving birth as causes Kunika

A few groups identified
Lack of awareness about Kunika
Lack of spousal communication about family planning
Ineffective family planning and
Lack of stigma around Kunika

2. What do you understand from what I have been telling you so far?

I am now going to show you some maps. We have drawn up these maps after consolidating maps from all the 26 groups.

< Facilitator shows the map on the most influential risk factors>

We generated this summary map from the maps of the 26 groups who had each drawn their own map about the causes of *kunika*. There are three main causes shown here, represented by these three coloured boxes (point to boxes). These are the three causes that came out as being the most influential causes of *kunika* according to the maps from all the groups. These lines (point to lines) show the links between these causes and *kunika*, either directly or indirectly. The thickness of the lines represents the strength of the links: the thickest lines mean the strongest influences.

The three causes shown in this map are:

Family Dynamics

Not using modern methods of family planning
Frequent sex and factors encouraging this

Family Dynamics includes polygamy-related jealousy and competition between wives, and specifically women wanting money to be spent in a naming ceremony. It includes a wish for a large family, by men and/or women, and desire for a child of a particular sex. It includes women wanting to complete their family quickly. Also social pressure and family pressure to have a large family. Sometimes women want to satisfy their husbands so that he remains faithful. It also includes monogamy, because the load (of satisfying the husband) falls to one wife.

The groups described many things leading to frequent sex, such as high desire, sharing a bed, husband being around, love and attraction, wearing make-up or tight clothes, sweet women, handsome men. Groups also mentioned use of sex-drive medicines, pornography, carelessness and impatience. They also included sexual contact while breastfeeding.

Not using modern methods of family planning includes general statements about "not using FP" or not using modern contraception. It includes not accepting modern FP methods, refusal to have CP injections, refusal to go to HF or HW for CP advice (both women and men). It also includes non-availability of FP commodities.

……………….

In each place separate groups of men and women created their own maps. There were some differences between men's and women's groups on their views about the causes of Kunika.

<Facilitator shows the map on men's views >

The men's map has the same factors as we just saw on the overall consolidated map.

<Facilitator shows the map on women's views >

The women's map has the same factors as the men's map but it also highlights the use of force or coercion as a cause of *kunika*.

3. Why do you think there is this difference between men's and women's groups on the causes of Kunika?
    a. Why did women's groups identify force or coercion as an important cause of Kunika?

……………….

The groups all made a second map on factors that reduce Kunika.

4. Do you remember some of the factors you mentioned when we made the map about prevention of kunika?

<Facilitator mentions the following>:
All groups identified
Use of modern methods of family planning
Reducing frequent sex

Almost all groups also mentioned

Better spousal communication about family planning
Improving family dynamics

Most groups also mentioned
Using non-invasive family planning
Use of health services and advice from health workers,

Many groups identified
Greater male involvement
Increase awareness about Kunika
Increase awareness about FP
Effective family planning
Prevention of forced sex
Create public awareness about family planning and Kunika

A few groups mentioned
Socio-economic conditions
Reducing fertility after birth
Increase stigma around Kunika
Ways of increasing FP awareness
Seek advice from traditional birth attendants
Belief and faith and fate

5. In your opinion, do these factors cover most of what you told us when we all made maps on Ba Kunika or prevention of kunika?

<Facilitator shows the map on the most influential protective factors >

The four most influential protective factors shown on this map are:

Create public awareness about family planning and Kunika
Use modern methods of family planning
Reduce frequent sex
Use non-invasive family planning methods

Creating public awareness includes making community notables and religious leaders aware about FP and kunika. It also includes increasing public awareness about kunika, including by groups such as community notables, health workers, religious leaders, CBOs. And targeting mothers, including in the curriculum, and media campaigns.

Using modern methods of family planning includes using various modern methods of family planning, and taking advice from health workers. It also includes increasing demand for family planning (eg by advice from HW), increasing availability of commodities, free distribution of FP commodities, and developing FP commodities with less side-effects.

Reducing frequent sex includes reducing related factors, such as suggestions about staying away from husband while breastfeeding, advice to avoid sex for 4-5 months after delivery and so on.

Non-Invasive FP includes use of condoms, calendar methods, withdrawal method, and traditional methods

……………….

There were some differences between men's and women's groups in maps on No Kunika.

<Facilitator shows the map on men's views >

Men's groups identified increasing public awareness about family planning and Kunika as an important way of reducing kunika.

<Facilitator shows the map on women's views >

Women's groups highlighted changing family dynamics as a way to prevent kunika.

Both men's and women's groups identified using modern methods of family planning as a way to reduce kunika. Women's groups also highlighted using non-invasive methods of family planning.

6. Why do you think there is this difference between men's and women's groups about way to prevent Kunika?

a. Why would men in particular highlight raising awareness about family planning and kunika?

b. Why would women in particular highlight use of non-invasive family planning methods and changing family dynamics?

……………….

Part 2

Action planning

In this session we will discuss ways to tackle issues regarding Kunika in communities.

1. A lot of men's groups told us that creating public awareness about family planning and Kunika will convince people to stop Kunika.

> 1a. In your opinion, what could be some of the ways by which we could create public awareness about family planning and Kunika in communities?
>
> 1b. Who should do it? <Ask for each suggestion>

2. One option for raising awareness would be through the home visits programme.

> 2a. In your opinion, how will people in households respond to us when we start discussing Kunika in our home visits?
>
> 2b. What are the kinds of things we have to be careful about when we discuss Kunika within households?
>
> 2c. Do we need to approach things differently with men and women in home visits?

3. Both men's and women's groups told us that using modern and non-invasive methods of family planning can stop Kunika.

> 3a. How can we convince men and women during our home visits to use modern and non-invasive methods of family planning?
>
> 3b. If you were a home visitor, what would you say to the man/ to the woman?

4. All men's and women's groups told us that frequent sex causes kunika. As we discussed there are several factors associated with frequent sex.

<Facilitator to ask the following questions separately for each of the factors listed below these questions.>

> > Can we discuss ………. with men and women at the household level?
>
> > If you were to discuss ……….. with men/women (use either as the type of group may be) in a household what would you say?

4a. high desire,

4b. sharing a bed,

4c. husband being around,

4d. love and attraction, wearing make-up or tight clothes,

4e. sweet women, handsome men,

4f. Use of sex-drive medicines,

4g. pornography,

4h. Carelessness and impatience,

4i. Sexual contact while breastfeeding.

5. Men's and women's groups told us that family dynamics play an important role in kunika. *Women's groups mentioned the role of family dynamics in stopping kunika. Again there are several factors associated with family dynamics.*

<Facilitator to ask the following questions separately for each of the factors listed below these questions.>

> Can we discuss ……… with men and women at the household level?

> If you were to discuss ……….. with men/women (use either as the type of group may be) in a household what would you say?

5a. polygamy-related jealousy and competition between wives, and specifically women wanting money to be spent in a naming ceremony

5b. a wish for a large family, by men and/or women,

5c. desire for a child of a particular sex

5d. women wanting to complete their family quickly

5e. social pressure and family pressure to have a large family.

5f. Women wanting to satisfy their husbands so that they remain faithful

5g. Monogamy, because the load (of satisfying the husband) falls to one wife

6. We are also planning to make a docudrama on what we learned from community maps on kunika include some of the issues related to family dynamics, frequent sex and use of modern and non-invasive family planning methods. We would show this drama to men and women during home visits.

6a. In your opinion, how could we show these issues in the drama?

6b. What could be the story?

6c. If you were an actor in the drama what would you say on each of these issues?

6d. Could I have two volunteers who enact what we just heard from the group for us today?
